# Outcomes in children treated with growth hormone for Prader-Willi syndrome: data from the ANSWER Program® and NordiNet® International Outcome Study

**DOI:** 10.1186/s13633-020-00090-6

**Published:** 2020-11-10

**Authors:** Moris Angulo, M. Jennifer Abuzzahab, Alberto Pietropoli, Vlady Ostrow, Nicky Kelepouris, Maithe Tauber

**Affiliations:** 1 NYU Winthrop Hospital, Mineola, NY USA; 2 Children’s Minnesota, St. Paul, MN USA; 3 Novo Nordisk Health Care AG, Zurich, Switzerland; 4 Novo Nordisk Inc., 800 Scudders Mills Rd, Plainsboro, NJ 08536 USA; 5 Center for Physiopathology of Toulouse-Purpan, and the Reference Centre for PWS, Department of Endocrinology, Children’s Hospital, CHU Toulouse, Toulouse, France

**Keywords:** Human growth hormone, Growth disorders, Body mass index, Body height, Registries

## Abstract

**Background:**

Growth hormone (GH) deficiency is common in patients with Prader-Willi syndrome (PWS) and leads to short adult stature. The current study assessed clinical outcomes based on real-world observational data in pediatric patients with PWS who were treated with GH.

**Methods:**

Data from patients previously naïve to treatment with GH who began therapy with somatropin were collected from 2006 to 2016 in the observational American Norditropin® Studies: Web-Enabled Research (ANSWER) Program® and NordiNet® International Outcome Study. Variables affecting change from baseline in height standard deviation scores (HSDS; *n* = 129) and body mass index standard deviation scores (BMI SDS; *n* = 98) were determined.

**Results:**

Patients included in both HSDS and BMI SDS analyses were treated with a mean GH dose of 0.03 mg/kg/d (SD, 0.01 mg/kg/d). Results from the HSDS analysis revealed that baseline age and years on treatment had a significant impact on the change in HSDS. In the BMI SDS analysis, longer GH treatment time led to a greater change in BMI SDS from baseline, and patients with a higher BMI at the start of treatment had a greater decrease in BMI over time.

**Conclusions:**

GH is effective in the management of children with PWS. Earlier treatment resulted in a greater gain in height, and a longer treatment period resulted in better outcomes for both height and BMI.

**Trial registration:**

This study was registered with ClinicalTrials.gov (NCT01009905) on November 9, 2009.

## Background

Prader-Willi syndrome (PWS) is a rare multisystemic genetic disorder that arises from the lack of expression of paternally inherited genes known to be imprinted and located in the 15q11-q13 region. In approximately 70% of cases, PWS is caused by a non-inherited deletion in the paternally derived chromosome in that region, while maternal disomy 15 causes 25% of PWS cases, and genomic imprinting defects cause 3% of cases [1]. The remaining 2% of cases are caused by rare translocations. The overall clinical picture of PWS is characterized by hypotonia, poor feeding in infancy, hyperphagia with evolving obesity, hypogonadism, decreased adult height, and cognitive and behavioral disabilities [1, 2].

More than 50% of infants and children with PWS are, or will become, GH deficient as determined by standard testing protocols [2]. Children with PWS who have GH deficiency do not experience height gain acceleration typical of puberty, leading to short adult stature (mean final height of 155 cm and 148 cm in males and females, respectively). Diminished growth can be observed as early as the prenatal period and can be sustained through infancy. Severe hypotonia is associated with feeding difficulties; however, short stature in PWS due to GH deficiency is unrelated to nutritional deficiency or hypothyroidism [1, 3].

Data on the successful use of exogenous human GH to improve linear growth in children with PWS were first published in 1987 [4] and were supported by subsequent investigations that showed sustained treatment with GH could improve growth, body composition, physical strength and agility, bone mineral density, fat utilization, cognition, and adaptive functioning [5–8]. Around the same time, recombinant human GH (rhGH) became available [9]. Approved by the US Food and Drug Administration (FDA) for the treatment of PWS in 2000 [3], rhGH demonstrated efficacy in increasing short-term growth and adult height in patients with a range of growth disorders [10–15]. The most recent clinical guidelines for the use of rhGH for PWS recommend that infants and children begin with a GH dose of 0.5 mg/m^2^/d, incrementally titrating every 3 to 6 months based on clinical response toward a dose of 1.0 mg/m^2^ [2].

Somatropin (Norditropin®; Novo Nordisk A/S, Bagsværd, Denmark) is an rhGH indicated for multiple conditions associated with GH deficiency, with an approved dose of 0.034 mg/kg/d for pediatric patients with PWS in the United States and Switzerland [16]. The non-interventional American Norditropin® Studies: Web-Enabled Research (ANSWER) Program® and the NordiNet® International Outcome Study (IOS) were long-term observational studies designed to assess real-life clinical outcomes of pediatric and adult patients treated with somatropin as prescribed by physicians according to standard clinical practice [17]. Data collected through both initiatives provided the opportunity to detail effects of GH treatment in specific diagnostic and demographic populations in the United States (ANSWER Program®) and other countries (NordiNet® IOS) [14, 18, 19]. The current study used data from both initiatives to assess clinical outcomes of pediatric patients with PWS treated with GH.

## Methods

### Data source

Data were extracted from the non-interventional ANSWER Program® and NordiNet® IOS, which aimed to evaluate long-term safety and effectiveness outcomes of pediatric and adult patients treated with somatropin in the United States and other countries, respectively. Details about both programs were described previously [17]. Briefly, the ANSWER Program® and NordiNet® IOS enrolled GH-naïve adults and children who were prescribed somatropin for treatment of growth disorders. Patient histories and physical examination data were entered by participating physician investigators using Web-based reporting forms. The doses of GH administered to participants were selected by treating physicians, and data on parameters such as baseline height standard deviation score (HSDS), weight, bone age, maximal stimulated serum GH concentration, serum insulin-like growth factor 1 (IGF-1) levels, and GH dose/frequency were collected [17]. Patient data from the ANSWER Program® were obtained over a 14-year period (June 24, 2002 to September 30, 2016) from 207 participating sites within the United States, while data from NordiNet® IOS were collected over 10 years (April 1, 2006 to December 31, 2016) from 469 clinics in 22 countries in Europe and the Middle East. The ANSWER Program® and NordiNet® IOS originally enrolled 20,204 and 17,995 pediatric patients, respectively [20].

### Assessments

Change from baseline in HSDS and body mass index standard deviation scores (BMI SDS) were analyzed using available follow-up data. Model-based least squares (LS) mean estimates of the impact of baseline parameters on change in HSDS and BMI SDS from baseline were determined using a repeated measures model analysis that included baseline HSDS, BMI SDS, gender, age, target height, region (United States/Europe), and GH dose. Outputs comprised LS mean changes from baseline at years 1 to 4, but statistical comparisons were not made.

## Results

### Patient characteristics

An initial total of 145 patients with PWS from the ANSWER Program® (*n* = 78) and NordiNet® IOS (*n* = 67) were eligible for inclusion for effectiveness outcomes assessments. Based on availability of follow-up data to perform the current analyses, a total of 129 patients were included in the HSDS analysis and 98 in the BMI SDS analysis. The mean ± SD GH dose administered to patients in both registries was 0.03 ± 0.01 mg/kg/d.

### HSDS analysis

Key demographic characteristics of the HSDS analysis are summarized in Table 1. Although proportions of males and females were balanced when combining both registries, taken individually, the ANSWER Program® and NordiNet® IOS provided higher proportions of females (59%) and males (60%), respectively. The overall mean (± SD) age at the start of GH treatment was 4.42 ± 4.51 years; however, patients were slightly younger in NordiNet® IOS (Table 1). The mean follow-up time was 2.48 ± 1.31 years and ranged up to 3.98 years.

**Table 1:** Demographic characteristics of patients for HSDS analysis^a^

Variable	ANSWER Program®	NordiNet® IOS	All
**Age at GH start, years**	*n* = 71	*n* = 58	*n* = 129
**Mean (SD)**	4.98 (4.73)	3.74 (4.18)	4.42 (4.51)
**Median (range)**	3.06 (0.15–17.56)	1.80 (0.39–15.81)	2.15 (0.15–17.56)
**Female, %**	59%	40%	50%
**Baseline BA/CA ratio**^**b**^	*n* = 15 1.04 (0.17)	*n* = 13 0.79 (0.31)	*n* = 28 0.92 (0.27)
**Final BA/CA ratio**^**b**^	*n* = 32 1.04 (0.18)	*n* = 27 0.88 (0.17)	*n* = 59 0.97 (0.19)
**GH baseline dose, mg/kg/d**	*n* = 71 0.03 (0.01)	*n* = 58 0.03 (0.01)	*n* = 129 0.03 (0.01)
**GH final dose, mg/kg/d**^**b**^	*n* = 61 0.03 (0.01)	*n* = 55 0.03 (0.01)	*n* = 116 0.03 (0.01)
**IGF-1 SDS**^**b**^	*n* = 31 −0.58 (1.79)	*n* = 34 − 0.84 (1.72)	*n* = 65 − 0.71 (1.75)
**Baseline height, cm**	*n* = 71 95.00 (33.47)	*n* = 58 88.75 (27.61)	*n* = 129 92.19 (31.01)
**Baseline weight, kg**	*n* = 71 24.65 (24.22)	*n* = 58 17.47 (18.56)	*n* = 129 21.42 (22.07)
**Last recorded height, cm**	*n* = 71 124.2 (31.14)	*n* = 58 122.1 (27.65)	*n* = 129 123.2 (29.52)
**Mid-parental target height, cm**^**b**^	*n* = 60 164.1 (25.33)	*n* = 38 174.5 (8.37)	*n* = 98 168.1 (21.04)
**Baseline HSDS**	*n* = 71 −1.17 (1.40)	*n* = 58 −1.94 (1.52)	*n* = 129 − 1.52 (1.50)
**Final HSDS**	*n* = 71 −0.28 (1.27)	*n* = 58 − 0.72 (1.11)	*n* = 129 − 0.48 (1.22)
**Years of follow-up**	*n* = 71	*n* = 58	*n* = 129
**Mean (SD)**	2.39 (1.33)	2.59 (1.29)	2.48 (1.31)
**Median (range)**	2.57 (0.06, 3.93)	3.10 (0.16, 3.98)	2.63 (0.06, 3.98)

*BA* bone age, *CA* chronologic age, *GH* growth hormone, *HSDS* height standard deviation score, *IGF-1* insulin-like growth factor 1, *SDS* standard deviation score

^a^All values mean (SD) unless otherwise specified

^b^Patient numbers reduced due to missing data

At baseline, mean height of patients in the HSDS analysis was slightly greater in the ANSWER Program® (95.0 ± 33.47 cm) compared with NordiNet® IOS (88.8 ± 27.61 cm); with a similar trend observed in mean BA/CA ratios (1.04 ± 0.17 and 0.79 ± 0.31, respectively; Table 1). At the end of follow-up, the final recorded heights of patients from the ANSWER Program® and NordiNet® IOS were 124.2 ± 31.14 cm and 122.1 ± 27.65, respectively; while the final recorded BA/CA ratios were 1.04 ± 0.18 and 0.88 ± 0.17, respectively.

**Fig. 1:**
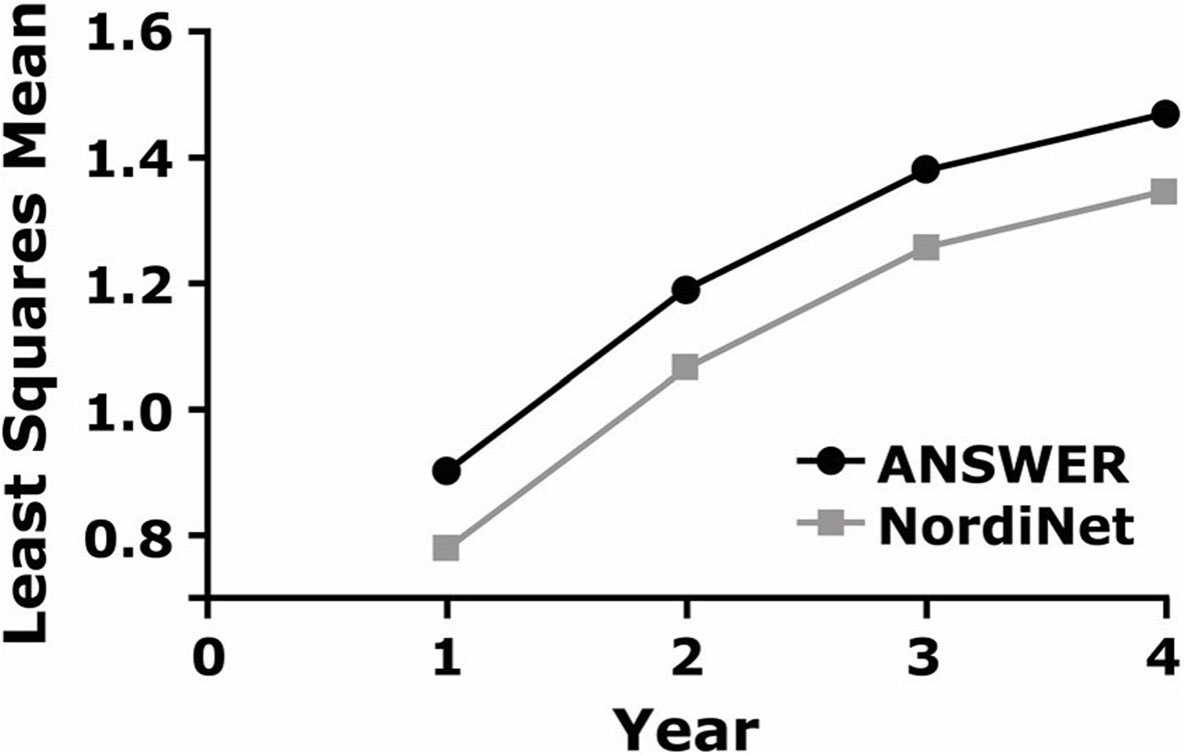
Model-based mean estimates for change from baseline over time in HSDS in patients with the diagnosis of PWS in both the ANSWER Program® and NordiNet® IOS. Abbreviations: HSDS, height standard deviation score; PWS, Prader-Willi syndrome

Mean baseline HSDS values were − 1.17 ± 1.40 and − 1.94 ± 1.52 from the ANSWER Program® and NordiNet® IOS, respectively. Results of the HSDS analysis revealed that baseline age (*P* = 0.014) and years on GH treatment (*P* < 0.0001) had significant impacts on changes in HSDS. Results from model-based mean estimates for change from baseline over time in HSDS are presented in Fig. 1. The findings demonstrated that patients in both the ANSWER Program® and NordiNet® IOS are expected to experience similar increases in HSDS over time.

### BMI SDS analysis

Key demographic characteristics of the BMI SDS analysis set are summarized in Table 2. At the start of GH treatment, mean patient age was 5.56 ± 4.79 years and mean duration of treatment was 2.45 ± 1.32 years. Baseline mean height of patients in the BMI SDS study was slightly greater from the ANSWER Program® (119.6 ± 25.24 cm) vs NordiNet® IOS (89.68 ± 28.29 cm), which was also reflected in mean baseline BA/CA ratios (1.06 ± 0.17 and 0.79 ± 0.31, respectively; Table 2). The last recorded heights of patients in the BMI SDS analysis from the ANSWER Program® and NordiNet® IOS were 140.3 ± 21.25 cm and 122.4 ± 27.55 cm, respectively; while the last recorded BA/CA ratios were 1.07 ± 0.17 and 0.88 ± 0.17, respectively.

**Table 2:** Demographic characteristics of patients for BMI SDS analysis^a^

Variable	ANSWER Program®	NordiNet® IOS	All
**Age at GH start, years**	*n* = 39	*n* = 59	*n* = 98
**Mean (SD)**	7.95 (4.21)	3.98 (4.52)	5.56 (4.79)
**Median (range)**	7.85 (2.04–17.56)	1.92 (0.39–17.66)	3.44 (0.39–17.66)
**Female, %**	62%	41%	49%
**Baseline BA/CA ratio**^**b**^	*n* = 13 1.06 (0.17)	*n* = 13 0.79 (0.31)	*n* = 26 0.92 (0.28)
**Final BA/CA ratio**^**b**^	*n* = 21 1.07 (0.17)	*n* = 27 0.88 (0.17)	*n* = 48 0.97 (0.19)
**GH baseline dose, mg/kg/d**	*n* = 39 0.03 (0.01)	*n* = 59 0.03 (0.01)	*n* = 98 0.03 (0.01)
**GH final dose, mg/kg/d**^**b**^	*n* = 35 0.03 (0.01)	*n* = 56 0.03 (0.01)	*n* = 91 0.03 (0.01)
**IGF-I SDS**^**b**^	*n* = 19 −0.46 (2.01)	*n* = 35 −0.87 (1.71)	*n* = 54 −0.72 (1.81)
**Weight, kg**	*n* = 39 38.89 (24.75)	*n* = 59 17.84 (18.61)	*n* = 98 26.21 (23.54)
**Baseline height, cm**	*n* = 39 119.6 (25.24)	*n* = 59 89.68 (28.29)	*n* = 98 101.6 (30.73)
**Last recorded height, cm**	*n* = 39 140.3 (21.25)	*n* = 59 122.4 (27.55)	*n* = 98 129.6 (26.61)
**BMI, kg/m**^**2**^	*n* = 39 24.68 (7.75)	*n* = 59 18.04 (4.90)	*n* = 98 20.68 (6.97)
**Baseline BMI SDS**	*n* = 39 1.80 (1.68)	*n* = 59 0.40 (1.54)	*n* = 98 0.96 (1.73)
**Final BMI SDS**	*n* = 39 1.88 (0.91)	*n* = 59 0.98 (1.83)	*n* = 98 1.34 (1.59)
**Years of follow-up**	*n* = 39	*n* = 59	*n* = 98
**Mean (SD)**	2.28 (1.34)	2.56 (1.30)	2.45 (1.32)
**Median (range)**	2.25 (0.06, 3.93)	3.09 (0.16, 3.98)	2.54 (0.06, 3.98)

*BA* bone age, *BMI* body mass index, *CA* chronologic age, *GH* growth hormone, *IGF-1* insulin-like growth factor 1, *SDS* standard deviation score

^a^All values mean (SD) unless otherwise specified

^b^Patient numbers reduced due to missing data

For the BMI SDS analysis, mean baseline BMI was lower in NordiNet® IOS (18.04 ± 4.90 kg/m^2^) than in the ANSWER Program® (24.68 ± 7.75 kg/m^2^). The same was true for baseline BMI SDS values: 0.40 ± 1.54 in NordiNet® IOS and 1.80 ± 1.68 in the ANSWER Program®.

Longer GH treatment time led to a significantly greater change in BMI SDS from baseline (*P* < 0.0001). When considering the interaction between baseline BMI and time, patients with a higher BMI at the start of treatment had a greater decrease in BMI over time (*P* < 0.0001).

**Fig. 2:**
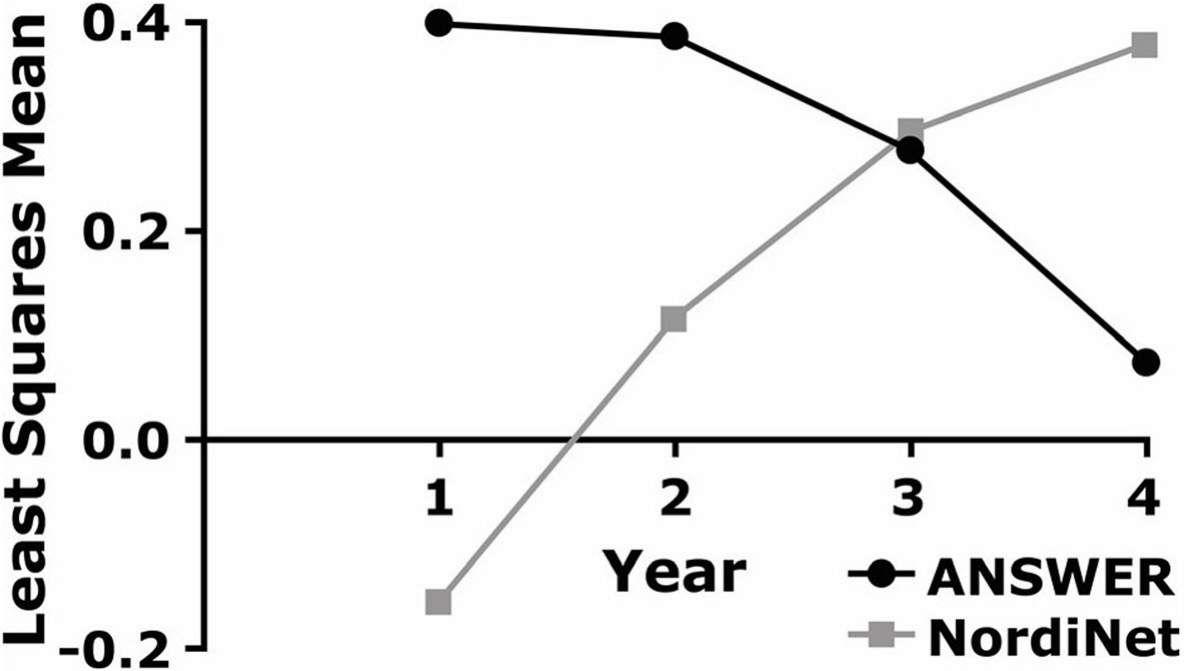
Model-based mean estimates for change from baseline over time in BMI SDS in patients with the diagnosis of PWS in both the ANSWER Program® and NordiNet® IOS. Abbreviations: BMI, body mass index; PWS, Prader-Willi syndrome; SDS, standard deviation score

Results from model-based mean estimates for change from baseline over time in BMI SDS are presented in Fig. 2. These results were obtained based on a model using a GH dose of 1.04 mg/m^2^, baseline age of 5.91 years, and baseline BMI SDS of 1.26. Notably, the expected change from baseline declined over time in patients from the ANSWER Program® but increased over time in patients from NordiNet® IOS. Using this model, the average hypothetical patient in the ANSWER Program® had an increase of approximately 0.4 kg/m^2^ in BMI SDS at year 1, followed by a return to baseline by year 4. The average hypothetical patient in NordiNet® IOS experienced an initial decline in BMI SDS of − 0.16 kg/m^2^ at year 1 followed by a gradual increase to 0.38 kg/m^2^ over baseline by year 4.

### Safety analysis

There were 8 non-serious adverse reactions and 0 serious adverse reactions reported in 6 patients with PWS in the ANSWER® registry. One death due to cardiopulmonary arrest was recorded; this occurred in a 2-year-old female patient and was considered unlikely to be related to treatment with somatropin. In the NordiNet® registry, 8 adverse reactions were reported in 6 patients with PWS, comprising 3 non-serious adverse reactions in 3 patients and 5 serious adverse reactions in 3 patients. Serious adverse reactions included type 1 and type 2 diabetes and scoliosis. In addition, 1 death of a 22-year old male was recorded; this sudden death was considered unlikely related to treatment with somatropin.

## Discussion

This analysis of real-world data from patients with PWS who initiated treatment with GH revealed that the treatment was effective in improving growth and body composition.

Improvement in linear growth following GH therapy in young patients with PWS is well established based on a long history of randomized clinical trials [21–32] and registry/observational studies, resulting in GH becoming the standard of care for PWS [8, 33–37]. Initiation of treatment with GH is recommended as early as possible at the time of diagnosis [37], and evidence supports continued use in adults to maintain bone health and promote lean muscle mass [38]. The current study contributes to the field by demonstrating that, in real-world conditions, treatment with GH initiated at an earlier age and continuing for a longer duration is significantly associated with improvements in HSDS compared with later initiation and shorter duration. The continued improvements over 4 years are consistent with previous studies demonstrating ongoing improvements in growth for up to 15 years of GH therapy [34–36, 39, 40].

Interestingly, in the BMI SDS analysis, results from our modeling studies showed opposite trends as BMI SDS increased and decreased over time for patients from NordiNet® IOS and the ANSWER Program®, respectively. This might be explained by the difference in the ages of patients in each registry, as the median age of enrollment (GH start) in the ANSWER Program® was 8 years (minimum 2 years) compared with 2 years (minimum 0.4 years) in NordiNet® IOS. Patients with PWS typically follow a series of age-dependent nutritional phases beginning with hypotonia and feeding difficulty up to 9 months of age, followed by normal feeding and growth until approximately 2 years of age when weight gain accelerates despite normal appetite. At approximately 4.5 years of age appetite increases, often leading to preoccupation with food and food-seeking behaviors that can result in obesity [41]. Due to this sequence of events, BMI SDS typically switches from below normal to above normal during the fourth year of life [42]; therefore, an increase or a decrease in BMI SDS could be considered an improvement, depending on the patient’s age and nutritional stage. As patients from the ANSWER Program® were, on average, older than those from NordiNet® IOS, it stands to reason that a larger proportion were in the insatiable, food-seeking phase compared with the younger patients in NordiNet® IOS. Also note that patients in the ANSWER Program® had higher mean baseline weight and BMI than patients in NordiNet® IOS, and similar age-dependent trends in BMI SDS in patients with PWS have also been observed in other studies [8, 33, 43–47].

Since the first demonstration over 30 years ago of the effectiveness of GH in improving growth in patients with PWS [4]. the use of GH has been widespread in these patients. The Global PWS Registry that includes approximately 2000 participants with PWS from 33 countries recently reported that 91% of patients are using (or previously used) GH, 71% of whom began treatment before 2 years of age. Of patients who used GH, 93% reported receiving benefits from the treatment [48, 49].

A notable strength of this study was the use of data from largest-to-date, real-world populations of patients treated with GH from NordiNet® IOS and the ANSWER Program®, totaling nearly 40,000 patients. However, by virtue of the size of these registries, data were collected at many sites by many different practitioners, presenting a likelihood of inconsistent data collection practices between sites, resulting in inconsistencies in patient numbers between parameters. Moreover, given that PWS is a rare disease, only a small overall number of patients was available for our analyses. Moreover, differences between NordiNet® IOS and the ANSWER Program®, such as the geographic/demographic backgrounds of patients, the aforementioned differences in patient ages, and the longer follow-up in NordiNet® IOS (median 3.1 years) vs the ANSWER Program® (median 2.25 years) may have produced volatility in the data and could underlie some of the differences in trends between registries, such as the different rates of change in height and BA/CA ratios. Finally, the study was limited more broadly by the general use of retrospective data.

## Conclusions

In conclusion, GH was confirmed to be effective in the management of children with PWS, and earlier treatment resulted in a greater gain in height, and longer treatment period resulted in better outcomes for both height and BMI. This study supports early initiation and sustained use of GH therapy in patients with PWS, which aligns with the current practice of starting treatment with GH before age 2 in toddlers with PWS to gain positive effects on growth and BMI [37].

## Data Availability

The data that support the findings of this study are available from Novo Nordisk, but restrictions apply to the availability of these data, which were used under license for the current study, and so are not publicly available. Data are however available from the authors upon reasonable request and with permission of Novo Nordisk, Inc.

## Abbreviations

BMI: Body mass index
GH: Growth hormone
HSDS: Height standard deviation score
IOS: International Outcome Study
PWS: Prader-Willi syndrome
rhGH: Recombinant human growth hormone
SDS: Standard deviation score
